# Biomimetic Protein Materials for Adjuvant-Free Dermal Penetration

**DOI:** 10.1021/acsmaterialslett.5c01250

**Published:** 2026-03-05

**Authors:** Marianna T. P. Favaro, Eric Voltà-Durán, Julieta M. Sánchez, Elvira Escribano-Ferrer, Vanesa Huaca, Eva Miranda-Tovar, Ugutz Unzueta, Eloi Parladé, Esther Vázquez, Ramon Mangues, Antonio Villaverde, Isolda Casanova

**Affiliations:** † Institut de Biotecnologia i de Biomedicina (IBB), 16719 Universitat Autònoma de Barcelona, 08320 Barcelona, Spain; ▲ Centro de Investigación Biomédica en Red de Bioingeniería, Biomateriales y Nanomedicina, Instituto de Salud Carlos III, 08034 Barcelona, Spain; § Departament d’Òptica i Optometria, Universitat Politècnica de Catalunya − BarcelonaTech, 08222 Terrassa, Spain; ∥ Instituto de Investigaciones Biológicas y Tecnológicas (IIByT), CONICET-Universidad Nacional de Córdoba, 5016 Córdoba, Argentina; ⊥ Departamento de Química, Cátedra de Química Biológica, Facultad de Ciencias Exactas, Físicas y Naturales, ICTA, Universidad Nacional de Córdoba, Av. Vélez Sársfield 1611, 5016 Córdoba, Argentina; # Department of Pharmacy, Pharmaceutical Technology and Physical Chemistry, School of Pharmacy and Food Sciences, University of Barcelona, 08028 Barcelona, Spain; ∇ Institute of Nanoscience and Nanotechnology (IN2UB), University of Barcelona, 08028 Barcelona, Spain; ○ 195999 Institut de Recerca Sant Pau (IR SANT PAU), 08041 Barcelona, Spain; ◆ Josep Carreras Leukaemia Research Institute (IJC), 08916 Badalona, Spain; ¶ Departament de Genètica i de Microbiologia, Universitat Autònoma de Barcelona, 08320 Barcelona, Spain

## Abstract

The effective dermal delivery of functional proteins could substantially improve therapeutic options for common skin disorders, in which current lipid-based or invasive strategies face efficacy and safety limitations. We report a biomimetic protein depot platform based on granular, nontoxic amyloids generated through Zn-mediated coordination of hexahistidine-tagged proteins. Functionalization of these materials with either the cell-penetrating peptide R9 or the tight junction modulator c-CPE, the C-terminal region of the *Clostridium perfringens* enterotoxin, enabled a systematic evaluation of transdermal penetration in mouse models. Whereas plain and R9-functionalized granules showed restricted permeation, c-CPE-functionalized granules achieved consistent distribution through the dermis into the hypodermal layers. These findings establish self-assembled protein amyloids as a promising and adaptable class of biomaterials for dermal protein delivery. Also, the ability of c-CPE to enhance permeability without auxiliary adjuvants, lipids, or invasive methods highlights the translational potential of this system for clinically applicable, noninvasive management of cutaneous conditions.

## Graphical abstract

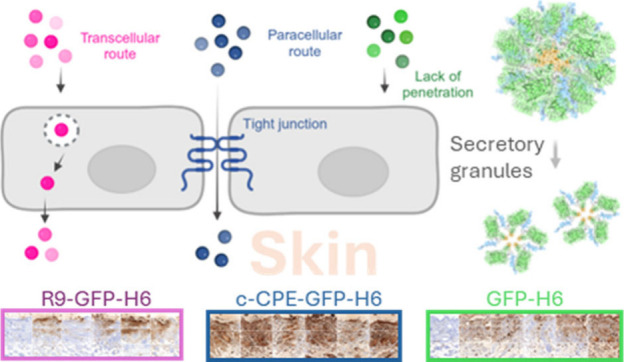

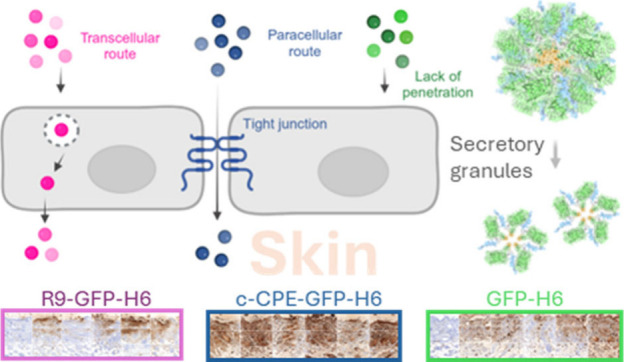

Skin is the major organ in the human body, and an architectonically complex and stratified biological barrier that restricts the penetration of environmental molecules and protects against pathogen invasion.^1^ This is due to its external corneal layer composed by dead cells, and its densely organized structure with cross-linked keratin filaments and regions rich in intercellular lipids.^2^ Favored by aging, which increases the fragility and vulnerability of this organ,^3^ many skin pathologies, including skin cancers, xerosis, venous and pressure ulcers, dermatitis, eczema, diabetic feet, itch, psoriasis, and a diversity of fungal, bacterial, and viral infections show a high prevalence,^4,5^ with a global burden and incidence expected to rise in the coming decades.^6–9^ The importance of achieving effective *in situ* skin delivery is reflected by the continuous exploration of improved pharmacological strategies.^13–15^ Drug delivery to the skin, commonly based on microemulsions^10,11^ and other colloidal systems,^12^ raises concerns regarding penetrability, stability, and efficacy, especially for hydrophilic compounds such as proteins. In this context, microneedles are efficient to overcome the stratum corneum barrier,^13–15^ but such an invasive approach limits patient acceptance. Less invasive, chemical penetration enhancers and/or on compositionally optimized drug nanocarriers are explored,^16^ but these approaches may be still insufficient and potentially toxic.^13–15,17–19^ Metal/metal-oxide nanoparticles, silica nanoparticles and quantum dots promote the release of reactive oxygen species and toxic ions,^20^ inflammation,^21^ and generic toxicities.^20^ Lipid nanocarriers are far less toxic than inorganic nanoparticles, but they still pose safety concerns linked to lipid composition, charge, and size.^22–24^ The risk of toxicity should not be overlooked even for topical administration, when used repeatedly over several weeks.

In this context, we have explored here the dermal penetrability of an emerging type of material for protein drug delivery, namely synthetic, functional amyloid-like particles that support an endocrine-like sustained release of the forming protein.^25–27^ Falling within the category of functional amyloids^28,29^ and mimicking secretory granules (SGs) from the mammal hormonal system,^30,31^ the protein, properly folded and biological active, is self-retained in microscale granular structures by means of the reversible coordination between ionic Zn and solvent-exposed histidine residues, overhanging from the polypeptide.^32,33^ Under physiological conditions, the building block protein is released in a time-sustained manner through Zn chelation. Being nontoxic in parenteral administration,^34,35^ the skin penetration capabilities of these granular materials have not yet been explored.^36–38^

For that, three alternative green fluorescent protein (GFP)-based proteins were selected for the study, namely, R9-GFP-H6, c-CPE-GFP-H6, and GFP-H6 (Figure 1A). Nonaarginine peptide (R9) is a cell-penetrating peptide (CPP) expected to promote transport by the transcellular pathway,^39^ while the C-terminal region of the *Clostridium perfringens* enterotoxin (c-CPE) is a relaxant of tight junctions (TJs)^40–42^ by the paracellular pathway (Figure 1B). Not being tested in transdermal delivery in intact skin models, these functionalities have been recently confirmed in plain soluble versions of these constructs.^43^ The proteins (including the control GFP-H6, where H6 is hexahistidine peptide) were produced as intact and highly pure (Table 1) soluble versions (Figure 1C,D). Proteomic data (Table 1) confirmed proteolytic stability and proper folding of GFP in all the constructs that remained fluorescent. Then, we selected Caco-2 cells as a well-established and accepted in vitro model to investigate molecular interactions involving TJ–associated proteins (such as claudins).^44,45^ Upon exposure (Figure 1E,F), the soluble c-CPE-GFP-H6 located in the intermembrane spaces, coincident with its reported TJ avidity,^43^ while R9-GFP-H6 predominantly accumulated as aggregates on the cell surface (possibly related to its high cationic character), although some fluorescence signals were consistent with intracellular localization. As expected, GFP-H6 showed no detectable membrane interaction (Figure 1E,F). The distinct subcellular distributions of the GFP signal confirmed that the appended peptides confer specific functionalities to the fusion proteins, underlying the different cell-interaction behaviors of the resulting constructs. Importantly, this feature might enable adaptation to clinical contexts in which either internalization-dependent or internalization-independent drug activity is required.

**Table 1:** Proteomic Properties of the Test Proteins

	theoretical MW (Da)	experimental MW (Da)	specific GFP fluorescence (%)^a^	mean hydrodynamic size at 37 °C (nm)	anti-GFP detection^b^	anti-H6-tag detection^b^	purity (%)
R9-GFP-H6	29,624	30,053	122.6 ± 0.3	8.36 ± 0.08	+	+	>95
c-CPE-GFP-H6	30,100	30,293	132.6 ± 0.6	6.41 ± 0.02	+	+	>95
GFP-H6	27,598	27,453	100 ± 0.3	5.29 ± 0.08	+	+	>95

a Relative to purified GFP-H6.

b Determined by Western blot.

**Figure 1:**
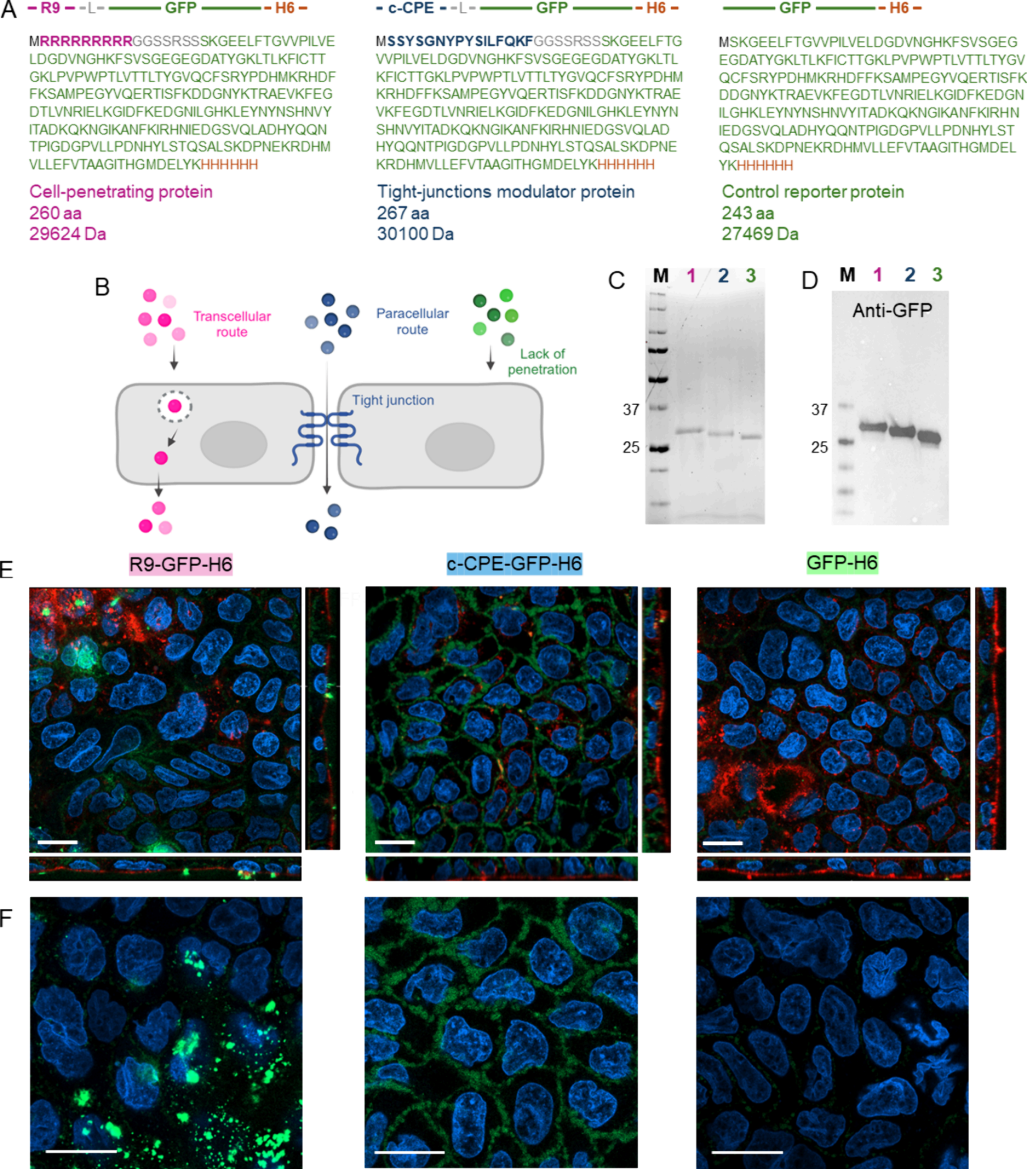
Modular proteins and cell penetration hypotheses. (A) Modular disposition of fluorescent proteins. GFP (in green) indicates the green fluorescent protein, H6 (in orange) represents a C-terminal hexahistidine, R9 (in pink) is a cell-penetrating peptide, L (in gray) is a peptide linker (GGSSRSS) for interdomain flexibility, and c-CPE (in blue) is the C-terminal domain of *Clostridium perfringens* enterotoxin. (B) Schematic pathways for the diffusion of proteins explored in this study. The transcellular route relies on the capability to penetrate membranes, whereas the paracellular route on the activity over tight junctions. (C) Analysis of protein purity and integrity upon bacterial production and purification through SDS-PAGE. (D) Immunodetection of GFP in blots. (E) Confocal microscopy images comparing the fluorescence intensity and distribution in a Caco-2 cell monolayer exposed for 1 h to soluble protein. Nuclei are stained with Hoechst 33342 (blue), GFP fluorescence is visualized in green, and membranes are stained with WGA-555 (red). (F) Single optical sections from the 3D reconstructions, showing the nuclei and the protein signal. Scale bars indicate 20 μm.

Once the analyses of soluble proteins were completed, SGs were constructed by Zn-mediated precipitation (Figure 2A), resulting in amorphous microparticles (Figure 2B). An amyloid-like architecture, inherent to such type of Zn-supported granular depots,^25,46^ was noted by the FTIR spectra (Figure 2C), with a dominant amide I component around 1625–1630 cm^–1^. This analysis evidenced a peak around 1630 cm^–1^, assigned to cross-β intermolecular organization of amyloid fibrils or aggregated β-rich structure^47^ and agrees with the observation over similar granular materials constructed with unrelated proteins.^25,48^ On the other hand, the Zn–His coordination, being reversible, should ensure the leakage of the forming protein from the granular material acting as dynamic depots.^49,50^ Protein leakage was confirmed in vitro in the three granular materials upon incubation in buffer for 3 days (Figure 2D), with almost complete SG disintegration. While this result does not necessarily reflect the temporality of protein release from the granules in vivo, it confirms the capability of the material to release its components. Interestingly, the released protein occurred in oligomeric, nanoscale forms as determined by DLS (Figure 2D, compare to the size of the monomers in Table 1), being this observation aligned with the concept of nanoparticles as intermediates in both formation and disintegration of SGs.^49^ In this regard, nanoscale oligomers leaked from comparable SG formulations in vivo preserve the same cell-targeting capability, interactivity, and uptake behavior observed in vitro,^25,34,35^ indicating that the Zn^2+^-mediated aggregation and release does not compromise the protein functionalities. As a further characterization step, we examined the penetrability of the leaked protein in HeLa cells, a robust and widely used reference for intracellular delivery and cytotoxicity studies.^51^ As observed, R9-GFP-H6 fluorescence was found to accumulate intracellularly in a time-dependent fashion (Figure 2E), upon a trypsin treatment designed to remove any protein that might be attached externally.^52^ This observation suggested that an important part of the granular protein is released in a soluble form under the cell culture conditions since the internalization of the whole granules is less plausible. In contrast, the R9-lacking proteins (GFP-H6 and c-CPE-GFP-H6) remained outside the cells, again confirming the cell-penetrating activity of R9 in this modular disposition. Any cell interaction of granules and leaked proteins with cultured cells occurred without loss of cell viability or evident toxicity (Figure 2F).

**Figure 2:**
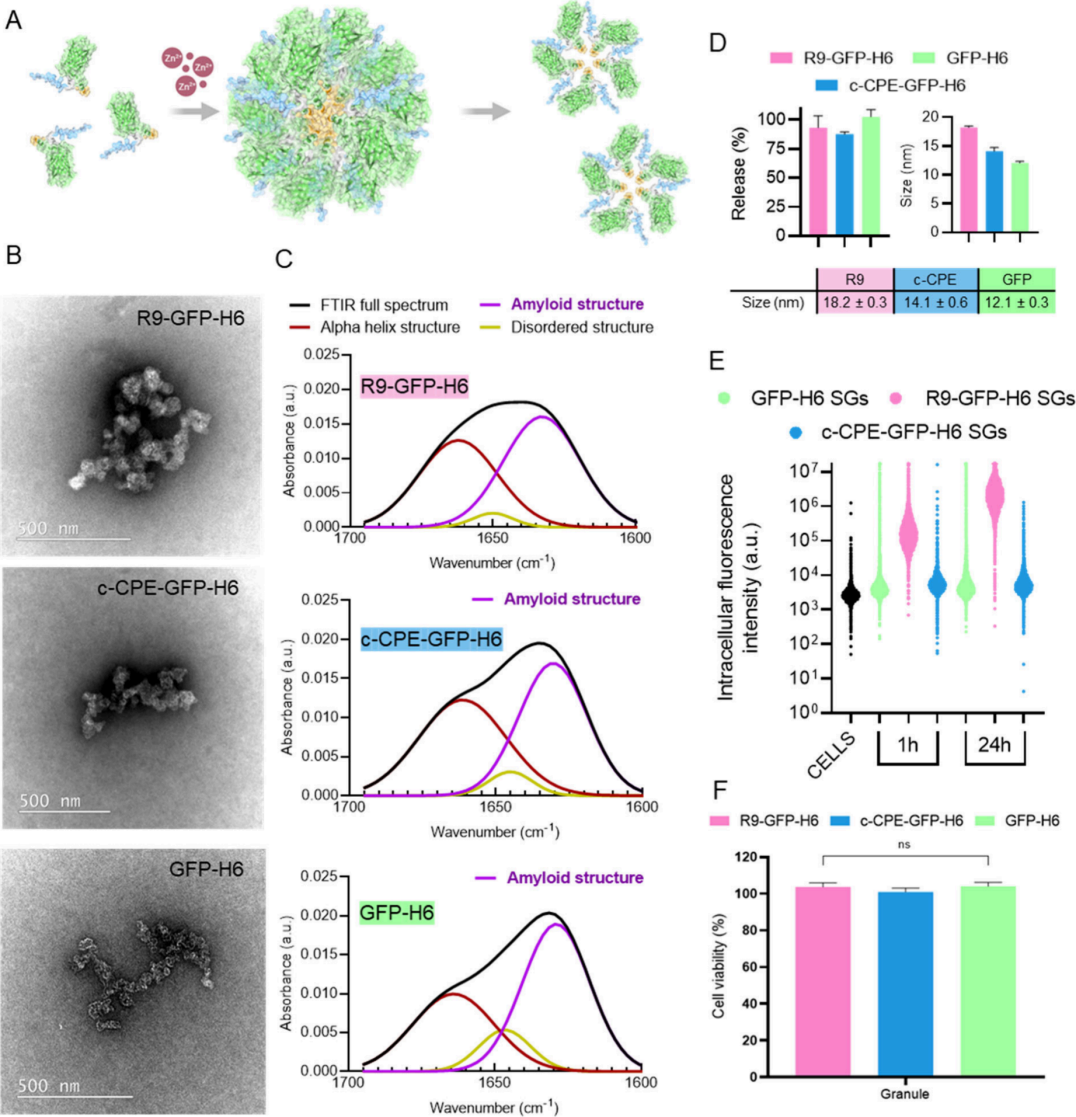
Principles, architecture, and *in vitro* performance of SGs. (A) Conceptual representation of Zn-assisted formation and progressive disintegration of artificial SGs. (B) TEM images of the SGs fabricated with the three model proteins. (C) FTIR spectra of the granular particles showing amyloid-like patterns. (D) Fraction of granular protein released at 3 days of buffer incubation (left) and size determination of the release protein (right). The numerical values are indicated at the bottom. (E) Intracellular GFP fluorescence in HeLa cells upon exposure to SGs for 1 and 24 h. (F) Comparative viability of HeLa cells upon exposure to granules at 1 μM for 48 h.

Based on these data, we proceeded to evaluate skin penetration of the granular materials in vivo using a nude mouse model. Since plain proteins typically cannot cross skin layers, we were particularly interested in determining whether our microscale platform could facilitate delivery into this organ. The granular protein versions were exposed to mouse skin loaded in patches (Figure 3A), and after 24 h, reporter GFP was immunodetected in histological samples at different levels, namely, epidermis (E), dermis (D) and hypodermis (H) (Figure 3B). In all the tested strata, differences between the study groups were observed (Figure 3B), with c-CPE-GFP-H6 being generically detected with much higher intensity than the other proteins. Also, the occurrence of c-CPE-GFP-H6 in the hypodermis was also more consistent when comparing alternative proteins (Figure 3B), indicative of a deeper penetration of c-CPE-functionalized materials. The presence of this construct in both superficial and deeper skin layers demonstrated that c-CPE was an effective tag for enhancing protein penetration into intact skin following topical application in an adjuvant-free platform. In contrast, R9 appeared to hinder tissue penetration, as skin biodistribution was more efficient for GFP-H6 compared to R9-GFP-H6 (Figure 3B).

**Figure 3:**
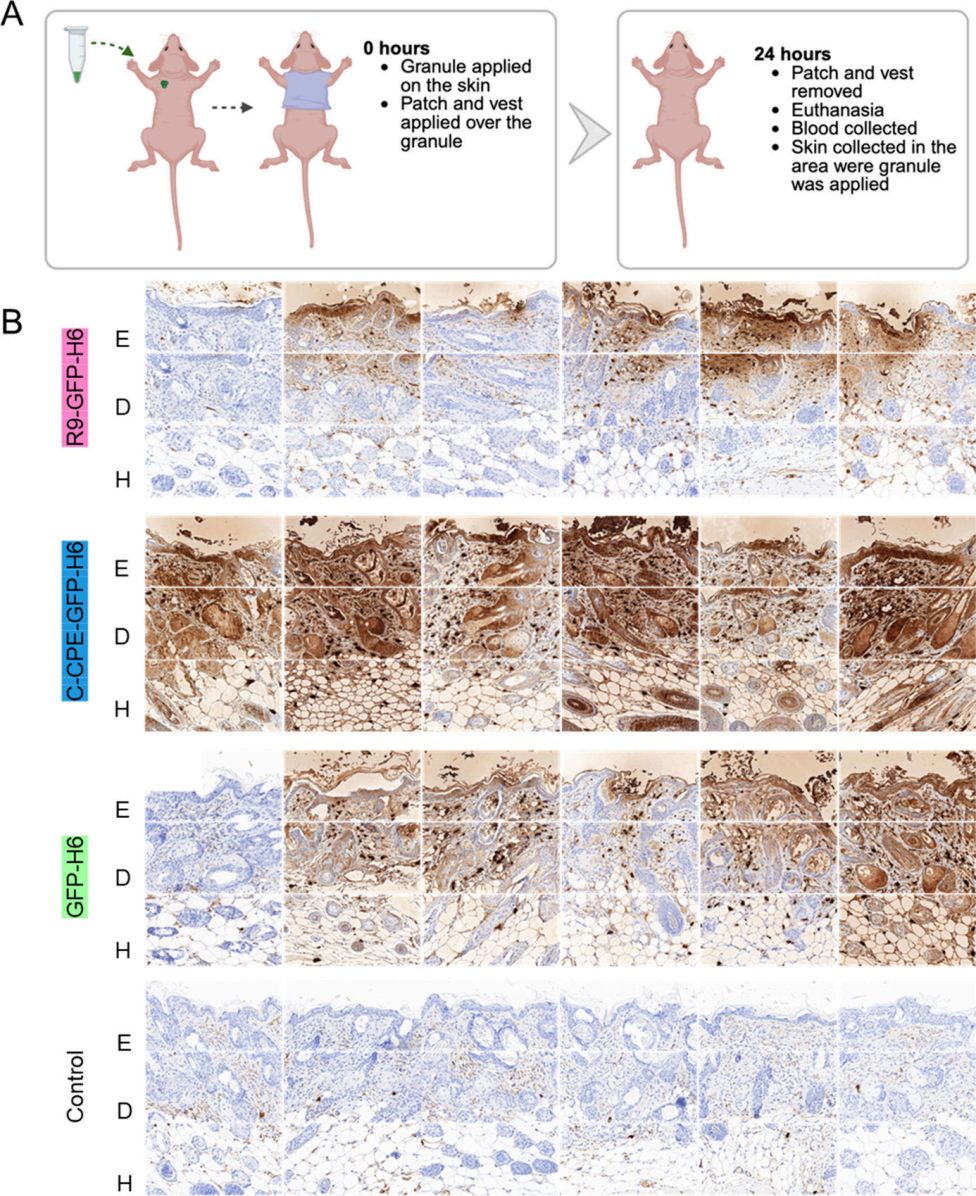
Topical application of protein granules. (A) Schematic representation of the application and further sampling. (B) *In situ* immunodetection of GFP upon sample collection, upon 24 h patch application. E indicates epidermis, D dermis, and H hypodermis. A total of six skin samples were analyzed per material type. The label “Control” indicates samples from untreated animals bearing empty patches.

For a refined comparison beyond visual analysis, the immunohistochemistry images were submitted to digital conversion to assess numerically the differences in tissue penetration. As envisaged, the variations regarding skin biodistribution were statistically proven, and c-CPE-GFP-H6 was confirmed as the most promising granular system (Figure 4A). In addition, the proteins did not enter the bloodstream in detectable amounts (Figure 4B), stressing the biosafety of the approach under the tested doses and conditions. Again, the deep localization reached by c-CPE-GFP-H6 placed this protein version as the most appropriate, among those tested, to reach a wide and deep skin distribution.

**Figure 4:**
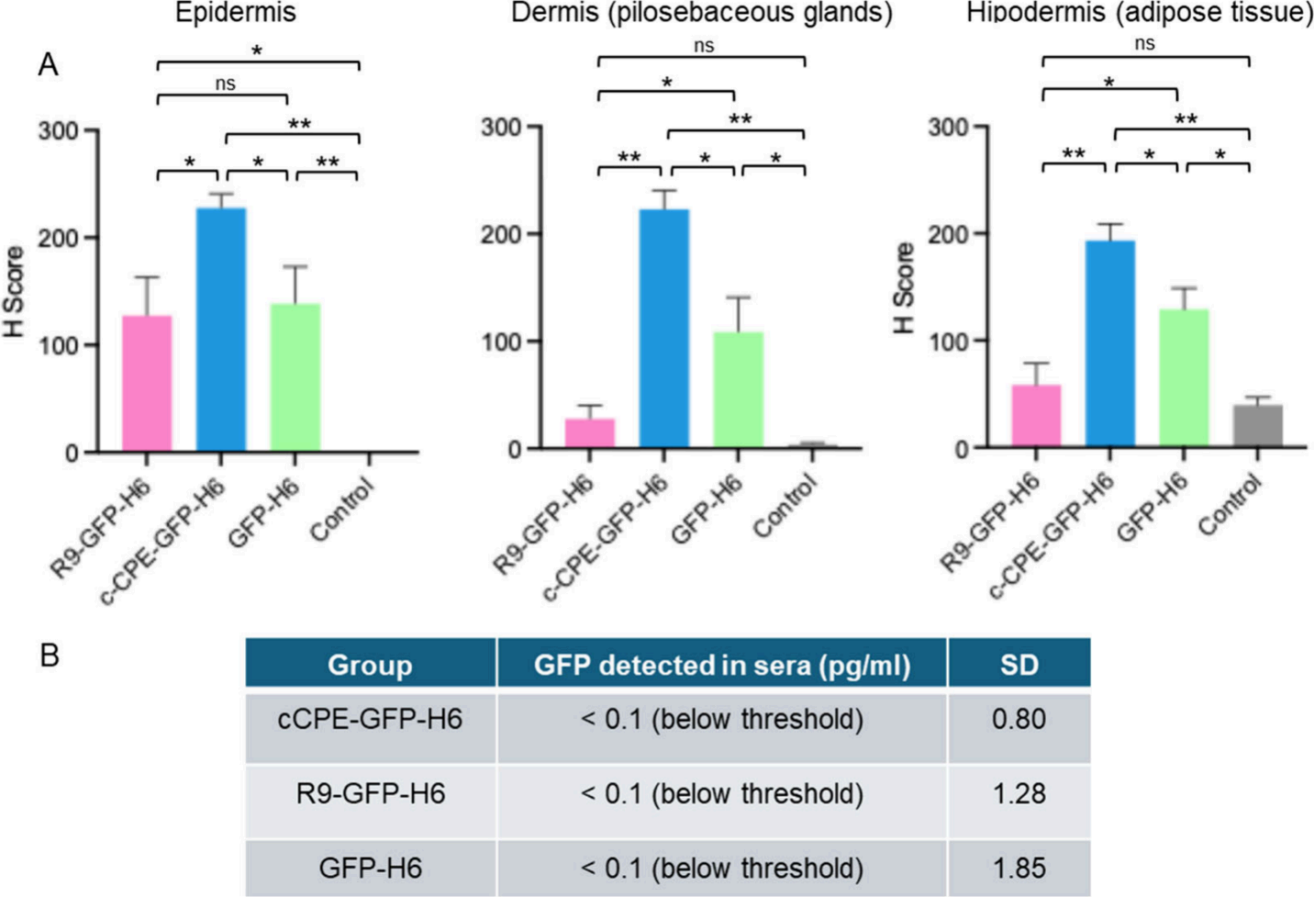
Skin penetration analysis of each protein. (A) Quantification of the protein at each layer on the immunohistochemistry images shown in Figure 3B, using Panoramic Scan II and DensitoQuant image analysis systems. The H score considers the intensity of signal in each area. Statistical analysis was performed using the Mann–Whitney *U* test. ** indicates *p* < 0.01, and * indicates *p* < 0.05. (B) Quantification of the protein reaching the blood, measured by anti-GFP ELISA on serum samples collected 24 h after the patch application.

Altogether, the results presented here indicate that artificial SGs are promising skin delivery systems for proteins (Figure 4). Notably, all the tested proteins showed a certain degree of skin penetrability (Figures 3 and 4), but the R9-empowered material and the nonfunctionalized GFP consistently failed to reach deep skin layers (Figure 4). Generically, CPPs, a category of protein segments to which R9 belongs, are expected to show potent skin-penetration abilities,^53^ although penetrability is irregular among CPP types and the underlying mechanisms are not well understood.^53,54^ R9, in our hands, has shown to be an excellent CPP when functionalizing GFP and nanoscale GFP oligomers.^43,55^ However, despite the initial hypothesis about its potential to drive tissue penetration of SGs (Figure 1), R9 failed to do so in the skin, resulting in moderate infiltration levels comparable to those of control nonfunctionalized GFP-H6 materials (Figures 3 and 4). In contrast, c-CPE, in the granular formulation, massively occurred in all skin layers, including epidermis, dermis, and hypodermis (Figures 3 and 4), indicative of its capability to penetrate and distribute into the tissue only upon 24 h of surface skin exposure. In a Caco-2 cell monolayer, c-CPE tended to attach to TJs,^43^ and *in vivo*, it acts as a powerful permeation enhancer (Figures 3 and 4). In this regard, c-CPE, primarily binding claudin 3 ^56^ and 4,^57^ enhances the permeability of diverse epidermal^58–60^ or mucosal^61^ models, but as far as we know, its skin penetration properties had not been examined in detail. In this context, when functionalizing SGs by genetic fusion (Figure 1), this peptide allows protein penetration into deeper skin strata (Figures 3 and 4) from surface-applied non-cytotoxic materials (Figure 2). The dissimilar effects of R9 and c-CPE indicate that these peptides are available for interaction and functionalities once they are exposed from granular depots. In this regard, the functionalization of the equivalent bacterial inclusion bodies (naturally produced in recombinant bacteria) formed by GFP with N-terminal fusion peptides (e.g., with cell surface protein ligands such as T22, binding the chemokine receptor CXCR4 ^37^) allows effective cell targeting, demonstrating the solvent exposure of the fused peptides in a significant fraction of protein microparticles.

At this stage, it is not possible to finely discriminate to which extent the skin-penetrating protein (Figures 3 and 4) is in granular form or in soluble version upon release. After subcutaneous administration, the chelation or dilution of gluing Zn^2+^ ions allows the time sustained disintegration of the granules for 1–2 weeks,^34^ linked to the progressive leakage of the biologically active polypeptides.^25,62^ While the extent and rate of disintegration have not yet been evaluated in dermal delivery, the occurrence of c-CPE-empowered material in the dermis and hypodermis and the analyses presented in Figures 1 and 2, favor the hypothesis of released soluble protein available at such levels. The presence of several skin enzymes, such as superoxide dismutase,^63^ which regulate Zn homeostasis through mild chelating activity,^64,65^ is expected to promote SG disassembly through Zn depletion. On the other hand, the undetected entry into the bloodstream would prevent systemic distribution (Figure 4B) and ensure the safety of this approach. Despite human and mouse skin differing in thickness, hair density, and attachment to the underlying tissue, both function as protective barriers with selective permeability and share key structural and functional features, including a stratified epidermis with a lipid-rich stratum corneum that governs permeation. These shared structures involve claudin-3 and claudin-4 to restrict the transit of ions and macromolecules using a paracellular pathway.^66,67^ Therefore, the data presented here in mice models are expected to be generically translatable to mammalian systems, including human.

A distinction is established between topical, dermal, and transdermal delivery routes.^68^ While transdermal systems focus on systemic exposure via dermal capillary uptake, clinical indications require penetration beyond the stratum corneum without systemic dissemination, such as local treatment of inflammatory skin diseases^69^ targeting immune cells in the epidermis and upper dermis, or intradermal vaccination and local immunomodulation strategies.^70,71^ In these contexts, controlled penetration and retention within the skin layers are preferable. The SG-based platform described here is inherently adaptable, as formulation parameters and functional tags can be modulated to favor topical, dermal, or transdermal protein delivery, providing a flexible framework to tailor the penetration depth and tissue localization. Through modular protein fusion (Figure 1), a broad repertoire of functional cargos can be incorporated, including viral antigens,^72^ growth factors,^27^ and antimicrobial peptides,^73^ the last ones particularly well suited for topical delivery via SGs,^74^ representing a promising clinical field.^75,76^ Beyond depot behavior that enhances topical penetrability, the SG format confers superior structural and functional stability compared to soluble proteins,^25,49,77^ potentially prolonging activity in complex skin environments. Distinct GFP localization in Caco-2 cells (Figure 1E,F) and internalization in HeLa cells (Figure 2E) further demonstrate that functionalizing peptides confer programmable, cell-specific interaction modes. Additionally, the Zn-supported granular system enables chemical conjugation,^78^ expanding payloads to protein-linked small molecules. Unlike other strategies,^79,80^ these self-contained self-delivered materials allow plain topical application without carriers, adjuvants, instrumentation, or invasive procedures, supporting simplicity and translational potential.

## Experimental Section

### Protein Production and Purification

Gene constructs were purchased from Geneart (Thermo Fisher) in pET22b (Novagen) and expressed in *E. coli* BL21 (DE3) (Novagen). Proteins were produced, purified, and characterized through standard procedures.^81–85^

### Analytical Methods

GFP fluorescence emission spectra were measured at 0.5 mg/mL protein in a Cary Eclipse spectrofluorometer (Agilent Technologies). The excitation slit was set at 2.5 nm, and the emission slit was set at 5 nm, with an excitation wavelength (λ _ex_) of 488 nm. Specific fluorescence was comparatively calculated as the fluorescence intensity at 512 nm relative to 1 mg/mL. Percentual values were calculated (considering 100% specific fluorescence of GFP-H6). Hydrodynamic size distribution of soluble proteins was determined by DLS at 633 nm and 37 °C in a Zetasizer Advance Pro (Malvern Instruments) instrument, measured in five replicates. The amyloid content within SGs was estimated by Fourier transform infrared spectroscopy (FTIR) in a Tensor 27 Bruker spectrometer with a Specac Golden Gate Attenuated Total Reflectance accessory, as described elsewhere.^25^ SGs were observed by transmission electron microscopy (TEM) by conventional procedures,^86^ through staining with 1% uranyl acetate (Polysciences Inc.) and observation in a JEOL 1400 transmission electron microscope (JEOL Ltd., Tokyo, Japan) with a Gatan Orius SC200 CCD camera (Gatan Inc. Abingdon, UK).

### Formation of SGs and Analysis of Protein Release

Recombinant histidine-tagged proteins and zinc chloride were mixed at 1:300 molar ratio (0.02 mM protein, 6 mM ZnCl_2_), in their respective storage buffers to obtain SGs.^49^ The release profile of soluble proteins from SGs was determined by resuspending the granules to a final concentration of 1 mg/mL in such respective storage buffers and incubating them at 37 °C for 3 days, when the material was centrifuged for 15 min at 15000*g* to collect the supernatant and to determine protein concentration by Bradford assay.

### Cell Culture

Standard cell culture procedures were applied in this study. Further details are given in the Supporting Information.

### *In Vivo* Assays

Female 10-week-old Swiss Nude mice from Charles River Laboratories (Wilmington, USA) were housed in a specific-pathogen-free (SPF) environment with sterile food and water provided *ad libitum* under 12 h light/dark cycles, and randomly assigned to one of four experimental groups (*n* = 6) (“Control” indicates absence of protein). A 1 mg dose of each SG material was deposited on the dorsal skin of each mouse and spread evenly by using a sterile loop (Deltalab) to enhance cutaneous absorption. The application site was then covered with a Durapore PVDF membrane (Merck Millipore), followed by a surgical dressing (Unidix) and sterile gauze to secure the patch and prevent mice from dislodging or ingesting it. The control group was processed in this way with no protein. After 24 h, the dressing and membrane were removed, and the mice were euthanized by cervical dislocation under isoflurane anesthesia. Following euthanasia, a skin section corresponding to the secretory granule administration area was excised and fixed in formalin for paraffin embedding. All permissions were obtained as indicated in the Supporting Information.

### Immunohistochemistry Staining

Protein penetration through the skin was quantified by anti-GFP immunohistochemical (IHC) staining. Paraffin-embedded skin samples were sectioned at 4 μm thickness and subjected to IHC staining using a DAKO Autostainer Link 48 (Agilent Technologies, Santa Clara, USA). Briefly, sections were dewaxed, and antigen retrieval was carried out using a low pH buffer (PTLink, Agilent Technologies). Subsequently, samples were stained on the DAKO Autostainer Link 48 using an anti-GFP primary antibody (1:400, sc-9996, Santa Cruz), and stained slides were scanned and quantified using the Panoramic Scan II and DensitoQuant image analysis system from 3DHISTECH Ltd. Representative images were acquired by using Slide Viewer software. The different skin layers (epidermis, dermis, and hypodermis) were quantified separately after manual definition of the corresponding areas. To quantify the dermis, only pilosebaceous glands were selected due to the high background present in the stroma. In addition, to quantify the hypodermis, only adipose tissue was considered. The H score values for each skin layer and mouse were automatically obtained with the image system after the definition of weak, medium, and strong staining intensities.

### GFP Detection by ELISA

GFP detection was performed on sera collected from animals at the end point of the experiment using the GFP ELISA kit (Abcam) following the manufacturer’s instructions.

### Statistical Analysis

Data processing and statistical analysis were performed on GraphPad Prism 9.4.0. Statistical significance was determined using two-way ANOVA or the Mann–Whitney *U* test as indicated in each figure. Differences between groups were considered statistically significant at *p* ≤ 0.05, represented by *.

## Supplementary Material



## Notes

Composition of storage buffers, precise experimental details of confocal microscopy, precise experimental details of protein internalization and cell viability assays, and statement regarding the approval and permissions for in vivo experiments. (PDF)

## Abbreviations

c-CPE: C-terminal region of *Clostridium perfringens* enterotoxin
CPP: cell-penetrating peptide
DLS: dynamic light scattering
FTIR: Fourier transform infrared spectroscopy
GFP: green fluorescent protein
H6: hexahistidine peptide
TEM: transmission electron microscopy
R9: nonaarginine peptide
SG: secretory granule
TJ: tight junction
