# Supplementary material for: Biomimetic Protein Materials for Adjuvant-Free Dermal Penetration
Source: ACS Mater Lett. 2026 Mar 5;8(4):1045–54. doi: 10.1021/acsmaterialslett.5c01250 (PMC13073729; doi:10.1021/acsmaterialslett.5c01250)
Supplement: Supplementary file 1 [file tz5c01250_si_001.pdf]

Biomimetic protein materials for adjuvant-free dermal penetration

Marianna T.P. Favaro [1,2‡], *Eric Voltà-Durán* [1,2,3‡], *Julieta M. Sánchez* [1,2,4,5], *Elvira Escribano-Ferrer* [6,7], *Vanesa Huaca* [2,8,9], *Eva Miranda-Tovar* [2,8,9], *Ugutz Unzueta* [2,8,9,10], *Eloi Parladé* [1,2,10], *Esther Vázquez* [1,2,10], *Ramon Mangues* [2,8,9*], *Antonio Villaverde* [1,2,10*], *Isolda Casanova* [2,8,9*]

[1] Institut de Biotecnologia i de Biomedicina (IBB), Universitat Autònoma de Barcelona, Barcelona 08320, Spain.

[2] Centro de Investigación Biomédica en Red de Bioingeniería, Biomateriales y Nanomedicina, Instituto de Salud Carlos III, Barcelona 08034, Spain

[3] Departament d'Òptica i Optometria, Universitat Politècnica de Catalunya – BarcelonaTech, Terrassa 08222, Spain

[4] Instituto de Investigaciones Biológicas y Tecnológicas (IIByT), CONICET-Universidad Nacional de Córdoba, Córdoba 5016, Argentina

[5] Departamento de Química, Cátedra de Química Biológica, Facultad de Ciencias Exactas, Físicas y Naturales, ICTA, Universidad Nacional de Córdoba, Av. Vélez Sársfield 1611, Córdoba 5016, Argentina

[6] Department of Pharmacy and Pharmaceutical Technology and Physical Chemistry, Pharmacy and Food Sciences School. University of Barcelona, Barcelona 08028, Spain

[7] Institute of Nanoscience and Nanotechnology (IN2UB), University of Barcelona, Barcelona 08028, Spain

[8] Institut de Recerca Sant Pau (IR SANT PAU), Barcelona 08041, Spain

[9] Josep Carreras Leukaemia Research Institute (IJC), Badalona 08916, Spain.

[10] Departament de Genètica i de Microbiologia, Universitat Autònoma de Barcelona, Barcelona 08320, Spain

Storage buffers. Upon protein purification in an Äkta Pure system (Cytiva) with 5 mL HisTrap HP columns (Cytiva), elution was based on a linear gradient of imidazole in an elution buffer (20 mM Tris-HCl, 500 mM NaCl, 500 mM Imidazole, pH 8.0), followed by serial dialysis cycles to remove imidazole. GFP-H6 was dialyzed against phosphate buffer (7.5 mM Na_2HPO_4, 2.5mM NaH_2PO_4 pH 7.4), c-CPE-GFP-H6 was dialyzed against phosphate buffer with 500 mM NaCl pH 7.4 and R9-GFP-H6 against phosphate buffer with 333 mM NaCl pH 7.4. These were the respective storage buffers.

Confocal microscopy. Caco-2 cells (human colorectal adenocarcinoma cancer cells, ECAAC) were maintained in Minimum Essential Medium, seeded in 8-well μ-Slide Grid-500 high iBiTreat (80806, Ibidi) plates at a density of 60,000 cells/cm^2 and grown for 3 weeks to form a tight epithelial monolayer, with media replacements every 2-3 days. On day 21, 10 μM of GFP-H6, c-CPE-GFP-H6 and R9-GFP-H6 were added in OptiPro™ SFM (Gibco) and incubated for 1 h. Then, protein was removed, cells washed with PBS and stained with Hoescht 33342 at 1 μg/mL and Wheat Germ Agglutinin – Alexa Fluor 555 (WGA 555, W32464, Invitrogen™, ThermoFisher Scientific) at 6.66 μg/mL in OptiProTM SFM. Images were obtained using a Zeiss LSM 980 confocal microscope (Zeiss) equipped with a 63x/1.4 oil immersion objective lens. Excitation was at 553 nm for WGA 555, at 488 nm for GFP and at 348 for Hoechst 33342. Emission was detected at 527-735 nm for WGA 555 (red), 490-546 for GFP (green) and 408-496 for Hoechst 33342 (blue). The WGA 555 detector operated at a gain voltage of 650V, the GFP detector at 620V and the Hoechst 33342 detector at 560-640 V. Images were acquired at different z-axis levels and processed using Imaris Viewer 9.5.1 to visualize the 3-dimensional distribution of proteins in the monolayer, as well as to collect the

images from a single layer as presented in Figure 1F. All images were equally acquired and processed using the same parameters.

Protein internalization and cell viability assay. HeLa cells (ATCC, CCL-2) were routinely cultured in Minimum Essential Medium (MEM Alpha Medium 1X + GlutaMAX™) (Gibco) supplemented with 10 % fetal bovine serum (Gibco) in a humidified atmosphere with 5 % CO_2 at 37 ˚C. Internalization assays were performed in 24-well plates with cells grown in their medium until 70 % confluence was reached, followed by a media exchange to OptiPRO Serum Free Medium before the addition of proteins. Protein (in SG form) uptake was determined at 1 h and 24 h at a final concentration of 1 µM. Cells were detached, and externally bound protein was removed by a harsh Trypsin-EDTA treatment (1 mg/mL exposure for 15 min at 37 ˚C). Intracellular protein fluorescence was determined by flow cytometry using a CytoFLEX (Beckman Coulter) with blue laser (488 nm), and fluorescence intensity was individually plotted for all the events collected.

For cell viability assays, HeLa cells were grown as previously described until transferred to opaque-walled 96-well plates to a final concentration of 3,500 cells per well and grown for 24 h until 70 % confluence was reached. Cells were exposed to 1 µM of each protein in the form of SGs. The cytotoxicity of the proteins was assessed after a 48-h incubation using the Cell Titer-Glo® Luminescent Cell Viability Assay (Promega) on a Victor 3 luminescent plate reader (Perkin Elmer). These experiments were conducted in triplicate, and the results were expressed as the percentage (%) of cell viability ± standard error.

Animal handling. Animal experiments were evaluated and approved by the Institutional Animal Care and Use Committee of the Sant Pau Research Institute and received authorization

from the Animal Experimental Committee of the local governmental authority (Generalitat de Catalunya, authorization No. 12123), in compliance with Spanish legislation (RD 53/2013) and the European Directive (2010/63/EU). All procedures were conducted at the Animal Experimentation Service, which holds ISO 9001:2015 certification. Furthermore, this study adheres to the Guide for the Care and Use of Laboratory Animals issued by the US National Institutes of Health (NIH Publication No. 85–23, revised 1985) and complies with the ARRIVE guidelines (Animal Research: Reporting of In Vivo Experiments).
